# Supplementary material for: Bifurcation, chaos, and stability analysis to the second fractional WBBM model
Source: PLoS One. 2024 Jul 23;19(7):e0307565. doi: 10.1371/journal.pone.0307565 (PMC11265713; doi:10.1371/journal.pone.0307565)
Supplement: Attachment — Submitted filename: comment-PONE-D-24-22626.docx [file pone.0307565.s001.docx]

**Manuscript Number:** PONE-D-24-22626

**Full Title:** Bifurcation, chaos, and stability analysis to the second fractional WBBM model

**By** Mohammad Safi Ullaha, M. Zulfikar Ali, Harun-Or Roshid

In this manuscript, the authors presented the bifurcation, chaos, and stability analysis for a significant the second 3D fractional Wazwaz-Benjamin-Bona-Mahony (WBBM) model in the research of shallow water waves. In addition to improving our understanding of shallow water nonlinear dynamics, including waveform features, bifurcation analysis, sensitivity, and stability, this study reveals insights into dynamic properties and wave patterns. The obtained results are interesting and novel. However, the following issues must be modified before it is considered for publication.

1. In Eq. (11), it must be emphasized that $l_{3}\neq l_{4}$. Otherwise, the bifurcation analysis mentioned below cannot occur.

2. For the literature on the Nizhnik Novikov Veselov model and various others, please introduce the latest achievements: Chaos, Solitons and Fractals 176 (2023) 114075 and Chinese Physics Letters 41, 044201 (2024).

3. In Eq. (8), to prevent singularities in future analysis, it is necessary to propose limiting conditions $l_{1}\neq0$, $l_{2}\neq0$, $l_{3}\neq0$, $l_{4}\neq0$.

4. On the next line of Eq. (12), it is mentioned that $\alpha=\frac{1}{l_{2}l_{4}}$, but Eq. (12) does not have this symbol at all. Please carefully check.

5. If Eq. (12) is $l$, the label in Fig. 1 must be modified, from $\alpha$ change to $l$.

6. After Eq. (12), all symbols must be carefully checked to prevent mixing of $\alpha$ and $l$.

In conclusion, if the author can make revisions according to the above suggestions, I recommend publishing this article in PLOS ONE.
